# 

**Published:** 1894-10-06

**Authors:** 


### The Hospital

AN INSTITUTIONAL JOURNAL OF

THE MEDICAL SCIENCES AND HOSPITAL ADMINISTRATION,

INCLUDING

A SPECIAL SUPPLEMENT FOR NURSES.

EDITED BY

HENRY C. BURDETT.

IN TWO VOLUMES ANNUALLY.

SURGEON GENL'S OFFICE, 152421. LIBRARY.

VOLUME XVII.

October to March, 1895.

London:

PUBLISHED BY THE SCIENTIFIC PRESS (LIMITED), AT THEIR OFFICE, 428, STRAND, W.C.

MDCCCXCV.

WI
H069X

### INDEX TO VOL. XVII., 1894.

*** Articles under the general heading *Medical Progress and Hospital Clinics* are distinguished by the initial (C.) for *Hospital Clinics*, and by (P.) for the rest of the series. (W.) distinguishes the *Institutional Workshop* articles, and (A.H.) the notes under the general title *Around the Hospitals*.

Abdomen, Tapping the (P.), 8, 25
Abdominal Sections (Methods) (P.), 443
Abdominal Sections, Thirst following (P.), 443
Abdominal Surgery (P.), 28, 443
Abscess (Neck) (P.), 187
Abscess, Cerebral (P.), 260
Acarophobia (P.), 81
Acetic Ether as a Stimulant (P.), 83
Acne (P.), 406
Aconitine, Poisoning by (P.), 226
Actinomycosis (P.), 64
Adelaide Hospital, Dublin, The (A.H.), 160
Adventures of a Tenth Century Medical Student, 272
After-care Association (W.), 302
Albuminuria and Life Assurance, 109
Alopecia (P.), 405
Anal Abscess, Superficial (P.), 365
Anal Condylomata (P.), 383
Anal Fissure (P.), 297, 365
Anal Tuberculosis (P.), 297
Anal Ulcer (P.), 365
Anal and Rectal Ailments, On Some Minor (C.), 365, 383
Anæsthetics (P.), 9, 26
Anæsthetics, Dangers of (P.), 10
Anastomosis Intestinal, A New Device (P.), 100
Anchylostomiasis (P.), 258
Aneurism, 415, 455
Aneurism (Surgery of) (P.), 11
Aneurisms (Neck) (P.), 188
Aneurisms, Origin and Classification of, 453
Annotations, *Under the various sub-titles.*
Annus Medicus, 1894, 233
Anthrax (P.), 64
Anti-Cholera Inoculation in India, 59 (*and see p.* 237)
Antipyretics (Infective Diseases) (P.), 423
Antiseptics and Surgery (P.), 312
Antitoxin. *See pp.* 219, 323, 434
Antitoxin in the London Hospitals, 205
Appendicitis, 38
Appendicitis and Appendicular Colic (P.), 171
Appendicitis Obliterans (P.), 330
Around the Hospitals (A. H.) (*See also under the names of the various hospitals*), 34, 69, 123, 160, 265, 320
Arsenic in Glycerine (P.), 156
Arthritis, Gonorrhœal (P.), 244
Arthropathy, Spinal (P.), 155
Asthenia, Cardiac (P.), 99
Ataxy (P.), 155
Australia, Hospitals in (W.), 87
Axitis, Chylous (P.), 331
Bacteria and their Products, 255
Baking. Does Baking Sterilise a Loaf? 77
Balfour. Mr. Balfour's Belief, 345
Barracks or Villages for Children, 59
Barrack Schools as Fever Hospitals, 360
Bedsteads, &c. (W.), 177, 227, 247, 264, 355
Bile Duct, Rupture of the Common (P.), 154
Biliary Lithiasis Treated with Ether (P.), 154
Bismuth Subnitrate, Poisoning from (P.), 244
Bladder, Paracentesis of the (P.), 25
Bladder, Surgery of the (P.), 242
Blackburn Fever Hospital (W.), 157
Blepharitis, The Treatment of (P.), 334
Blind, Institutions for the (W.), 228
Blind. The Post Office and the Blind (W.), 282
Boarding Schools as Disseminators of Disease, 341 (*and see p.* 386)
Boils, The Treatment of, 149
Book World of Medicine and Science, The (books, magazines, and pamphlets reviewed or noticed, and articles relating to)—
  Blake. Myxœdema, Cretinism, and the Goitres, 285
  Boies. Prisoners and Paupers, 231
  Brodie. Dissections Illustrated (Pt. III.), 267
  Bury. Clinical Medicine, 125
  Cheyne. The Treatment of Wounds, Ulcers, and Abscesses, 161
  Church Magazine, A New (The Minster), 161
  Cooke. Edible and Poisonous Mushrooms, 197
  Darwin. Practical Physiology of Plants, 339
  Dembo. Jewish Method of Slaughter, 107
  Despagnet. *See Nimier*
  Diary. The City Diary, 267
  Doses and Strength (Middlesex College of Chemistry), 89
  Drewth. Gangrene of the Limbs following Typhoid Fever, 197
  Duane. Student's Dictionary of Medicine, &c., 449
  Ellis. Man and Woman, 17
  Fayrer. On Preservation of Health in India, 411
  Fick. Lehrbuch der Augenheilkunde, 357
  Green. *See Muller*
  Hall. Diseases of the Nose and Throat, 267
Book World of Medicine and Science (*continued*)
  Hamilton. Text-book of Pathology, 303
  Herman. Difficult Labour, 53
  International Clinics (Quarterly Review), 89
  Kelly's London Medical Directory, 375
  Kipping. *See Perkin*
  McNeill (R.) The Prevention of Epidemics, 431
  Mercier. Lunacy Law for Medical Men, 267
  Muller (Thomson). Spinal Curvature and Awkward Deportment, 321
  Muskett. Prescribing and Treatment in the Diseases of Infants and Children, 71
  Nimier and Despagnet. Traité Elémentaire d'Ophthalmologie, 357
  Onodi (R. Green). Anatomy of the Nasal Cavity, 357
  Our Dogs, 375
  Parker. Post-nasal Growths, 339
  Perry. *See Shaw*
  Perkin and Kipping. Organic Chemistry, 71, 375
  Photographic Quarterly Review, 89
  Pocket Therapeutic Notes, 339
  Quarterly Journal of the Sanitary Institute, 35
  Riddell. A Manual of Ambulance, 467
  Roberts. Theory and Practice of Medicine, 339
  Roose. Gout and its Relations to Diseases of the Liver and Kidney, 17
  Science Progress (Vol. I.), 143
  Shaw and Perry. Catalogue of Pathological Specimens in the Museum of Guy's Hospital, 285
  Swanzy (Ed. Werner). Handbook of Diseases of the Eye, 357
  Thomson. *See Onodi*
  Tuke. The Insanity of Over-exertion of the Brain, 107
  Vacher. Defects in Plumbing and Drainage Work, 249
  Werner. *See Swanzy*
  Whitehead. Fruit Culture for Profit, 197
  Withington. Medical History from the Earliest Times, 125
Breast, Tumours of the (P.), 242
Bromide of Ethyl (P.), 27
Browne's (Sir J. C.) Cure for Quackery, 55
Bryant (Mr. T.), on the Public and the Profession, 41
Bubonic Plague (P.), 46
Bulbar Palsy, Unilateral (P.), 173
Burdett (Mr.), Address by, 228
Burning Questions, 215
Bursæ, Enlarged (Neck) (P.), 188
Cactus Grandiflorus, A Study in Therapeutics (P.), 62, 444
Calculus, Renal (P.), 227
Canadian Hospitals (W.), 67, 122
Cancer in the Rectum, The Early Diagnosis of, 146
Carcinoma, Resection for Pyloric (P), 66
Carcinoma, Pathology of (P), 188
Cardiac Tonics (P.), 442
Cataract, Laminar, 3
Catarrh. *See Ear*
Catarrh, Infectious, 345
Catheterisation. *See p.* 115
Cerebellum. Efferent Fibres from the (P), 173
Cerebral, Abscess (Chronic) (P.), 441
Cerebral Surgery, The Possibilities of, 149
Cervix during Menstruation, The (P.), 352
Channels of Infection, 216
Chapman, Dr. John (Obituary), 174
Charnwood Forest Convalescent Home (W.), 13
Chelsea Hospital for Women, The, 37, 50, 159, 310, 328
Children, Diseases of (P.), 275 294, 311
Children's Cots. *See Bedsteads*
Chloroform, Administration of (P.), 11
Chloroform and Salicylide Chloroform (P.), 9, 10
Chloroform in Organic Heart Disease (P.), 10
Cholera (*and see p.* 59) (P.), 47, 63
Cholera Inoculation: More Evidence, 237
Cholesteatoma of the Ear (P.), 425
Christmas Charity, The Flowing Tide of, 199
Churchill, Lord Randolph, The Memory of, 309
Circulatory System, Diseases of the (P.), 441, 459
Cirrhosis of the Liver (P.), 241
Cirrhosis, Hepatic, with Enlarged Spleen (P.), 241
Climate and Death-rate, 437
Clinical Society, The, 41
Clinical Society, The: Discussion on Antitoxin, 219
Club-foot (P.), 349
Club-foot, Bone Operations for (P.), 349
Club-foot: Its Nature and Treatment (C.), 401
Coal Tar, Compound Tincture of (P.), 298
Coccydynia (P.), 262
Codeine, Uses of (P.), 297
Cold, Healthy and Unhealthy, 397
Colic, Hepatic, Treatment of (P.), 242
Commercialised Physic: A Protest, 437
Communism or Christianity. (A Story of the Future) 211
Compulsory Removal to Hospitals, 167
Conculcation or Inoculation, 251
Concussion, Cranial (P.), 260
Construction Notes (W.), 52, 69, 105, 159, 178, 248, 266, 466
Consumption, The Drug Treatment of, 74
Consumption, Heredity in, 288
Consumption, The Plague of (C.), 457
Convulsions, Infantile (P.), 275
Cornea, Diseases of the, 343, 417
Cornea, Lactic Acid in Ulcers of the, 334
Correspondence. [*Editor's Letter Box*]
County Council, London Hospitals and the (W.), 317
Criminal Responsibility in the Insane, 19
Craig House Asylum, Edinburgh, the New (W.), 86
Cranial Vault, Œsteoporosis of (P.), 261
Craniectomy (P.), 259
Craniotomy in Microcephalus (P.), 295
Cycling and Heart Disease, 273
Cystitis (C.), 169
Cystitis, Chronic (P.), 458
Cysts (Neck) (P.), 187
Dampness and Rheumatism, 437
Dartford, The Livingstone Cottage Hospital (W.), 353
Darwinism and Race Progress, 344
Deadly Disease of Our Own Times, A, 395
De Motu Cordis et Sanguinis, 255
Derbyshire Royal Infirmary (W.), 335, 354, 408
Dermatitis Herpetiformia (P.), 368
Diabetes, Diet Complications, &c. (P.), 385
Dermatology, Periscope of (P.), 81, 101 (*and under various sub-titles*)
Dermatology, Therapeutics of (P.), 82, 406
Dermatology, Miscellaneous Notes (P.), 82
Dermatology, Progress in (P.), 367, 405
Devon and Exeter Hospital (W.), 103, 427, 448
Diarrhœa, Summer (P.), 258
Diarrhœa, Guava in (P.), 258
Diet. The Right Diet for John Bull, 419
Diphtheria (Note), 401
Diphtheria, The Antitoxin Treatment of, 182
Diphtheria: "The Brute Force of Figures," 205
Diphtheria, The Trade in Remedies for, 131
Diphtheritic Paralysis in Children (C.), 239, 257
Divorce between Medicine and Surgery, The, 163
Doctors and the Midwives, The, 205
Doctors and Relieving Officers, 77
Doncaster Infirmary and Dispensary, The (A.H.), 265
Drink without Drunkenness, 309
Drugs. *See New Drugs, &c.*
Drunkenness, "Cures" for, 113
Dry Air (Heated), Use of in Surgery (P.), 348
Dry Sanitation, 360
Duel, A Medical, 202
Dulcin (P.), 83
Duodenum, Perforating Ulcer of (P.), 119
Duodenum, Large Pseudo-Diverticulum of the (P.), 120
Duodenum, Pseudo-Diverticulum of the (P.), 330
Dysentery (Etiology; Treatment) (P.), 258
Ear. *See Otology*
Ear, Chronic Catarrh of the Middle Ear (C.), 115, 133
Eastbourne, All Saints' Convalescent Hospital (W.), 175
Eastbourne, Princess Alice Hospital (W.), 121
Eclampsia (Puerperal Temperature) (P.), 152
Editor's Letter-box, 16, 70, 84, 106, 124, 138, 148, 196, 230, 248, 298, 370, 386, 411, 448
Education and Disease, 270
Eight Hours Day (*see p.* 308)
Eczema (P.), 267
Electric Lighting for Hospitals (W.), 68, 105, 123
Electricity, the Dangers of, 452
Empyema (Surgery in) (P.), 7
Empyema in Children (P.), 296
Endocarditis (P.), 459
Enteric Fever in Infancy (P.), 312
Enteroplasty (P.), 120
Enterectomy for Rupture of the Ileum (P.), 119
Enterography Circular (P.), 120
Epilepsy (P.), 190, 260
Epilepsy, Infantile (P.), 275
Epileptics, New Institution for, in Berlin, 49
Episcopal Surgeon, An, 148
Ergot for Fibroids (P.), 350
Erysipelas (P.), 368
Eruptions Due to Drugs (Dermatology) (P.), 82
Ether (P.), 26
Ethical in Modern Medicine, The, 377
Factories and Workshops Bill, The, 399

Fæcal Concretions (P.), 330
Falmouth Cottage Hospital (W.), 193
Febrile Disturbance in Women. Some Causes of, during the Lying-in Period (C.), 151
Feet. Sweating Feet and Flat Feet (P.), 350
Femur. Bending of the Neck of the Femur in Adolescence (P.), 350
Ferratin (P.), 262
Fever Hospitals, The Planning of (W.), 445
Fevers. Infectious (P.), 275
Field of Science, The, 76
Filter, The Domestic, 237
Financial Strength of our Voluntary Charities (W.), 245, 263, 281, 299
First Century of English Medical Literature, 40, 112, 166
Fistula (Rectal Surgery), (P.), 278
"Flat Men," 309
Football Players, Impetigo (?), 399
Foreign Hospitals (W.); 104, 336
Fractures and Dislocations (General Surgery) (P.), 332
Friedreich's Disease, Non-hereditary (P.), 155
Gall-bladder, Operations on the (P.), 153
Gangrenous Bowels, Resection of (P.), 101
Gastric Ulcer Associated with Perforation. Operative Treatment of (P.), 65
Gastronomy, 200
Gastronomy for Dilatation of Œsophagus (P.) 65
Gavage or Forced Feeding (P.), 312
General Medical Council, The, 191
Glandular Swellings (Neck) (P.), 203
Glasgow's Hospital Sunday, 113
Glaucoma, A New Operation for (P.), 333
Gonorrhœal Arthritis (P.), 244
Great Northern Central Hospital, The, 6, 138, 141, 195, 363, 372, 387, 389
Growths. New Growths (Dermatology) (P.), 82
Growths, Malignant (C.), 221
Growths of the Rectum (P.), 278
Guaiacol as an Antipyretic (P.) 118
Guaiacol, Poisoning by (P.), 352
Gunshot Wounds (P.), 261, 333
Guy's Hospital (A.H.), 34
Gynæcology, Progress in (P), 352
Hæmorrhoids (Treatment) (P.); 296
Hallux Rigidus (P.), 349
Harveian Oration, The, 66
Head Injuries (C.), 329
Health by Act of Parliament, 413
Heart, The Senile, 73 (*and see p.* 419)
Heart and Vascular System, Diseases of (C.), 79, 99
Heating and Ventilation (W.), 408, 446
Hemiplegia, On (C.), 221
Hernia, Gangrenous, Strangulated, 366; Latent, Vesical, &c. (P.), 404
Hernia and Herniotomy (in Children) (P.), 295
Hip, Operative Treatment for old Unreduced Dislocations of the (P.), 350; Congenital Displacement of the (P.), 350
Holmes (Dr. Wendell), Death of, 23
Hong Kong Hospitals (W.), 319
Hospital Administration (W.), 193, 317, 335, 354, 372, 387, 408, 463
Hospital Construction (W.), 13, 49, 85, 157, 193, 319, 353, 407
Hospital Festivals and Meetings (W.), 16, 50, 142, 159, 195, 228, 284, 301, 338, 355, 373, 391, 409, 428, 465
Hospital Finance (W.), 31, 371, 427
Hospital Re-construction (W.), 427
Hospital Sunday. Who were its Founders and Originators? 32
Hospital Sunday Fund, Metropolitan (W.), 228
Hospital Sunday, Glasgow's, 113
Hunter (John) as a Biologist, 381
Hydrastis Canadensis (Therapeutics) (P.), 83
Hydronephrosis (P.), 277
Hygiene in Schools, The Teaching of, 5
Hyperpyrexia, the Genesis and Treatment of, 127
Hypochondriasis (note), 24
Hysteria (P.), 191
Hysterical Knee, A Case of (P.), 44
Hypertrophy of the Prostate, 164, 460
Hypertrophy, Idiopathic, of the Heart (P.), 100
Icthyol Collodion (P.), 174
Idiots, Imbeciles, and Feeble-minded Children, The Medical Care of, 454
Immunisation, Experiments in, 185
Immunity from Infective Diseases (P.), 423
India, Letters from, 316
Inebriates, Institution for (Walnut Lodge), (W.) 227
Infantile Scurvy, 95
Infectious Diseases: The Fruits of Notification, 381
Infective Diseases (P.), 46, 63, 117, 223, 403, 423
Infective Diseases, General Pathology of (P.), 117
Infective Diseases, Immunity from (P.), 423
Influenza (P.), 403
Influenza Convalescents, 399
Influenza: Do the Doctors Know Anything About it? 381
Influenza, Ordinary and Extraordinary, 419
Ingrowing Toenail and its Radical Cure (C.), 61
Inheritance, 92
Insane. *See Story of the Insane*
Insane, The Disposal and Treatment of the, 326
Insane Disorders of Childhood (P.), 312
Insanity. Is Insanity Increasing in Scotland? 451
Insanity and Inoculation (note), 204
Intestinal Anastomosis, A New Device for (P.), 100
Intestinal Approximation (P.), 100
Intestinal Decomposition and Milk Diet (P.), 330
Intestinal Diseases (P.), 258
Intestinal Diseases: Kraske's Operation (P.), 279
Intestinal Obstruction, The Treatment of, 131
Intestinal Perforation (P.), 119
Intestinal Putrefaction (P.), 330
Intestine. How much of the Intestine may be Excised? (P.), 100
Intestines, Diseases of the (P.), 330
Intra-Uterine Cauterisation (P.), 351
Intussusception, Treatment of, by Laparotomy (P.), 120
Iodide of Potassium in Human Actinomycosis (P.), 370
Ireland for a Holiday, 23
Iris, Inflammation of the, 39, 111, 183, 271
Iritis. *See Iris*
Isolation Hospitals as Centres of Infection, 359
Japanese as Experimental Physiologists, 23
Jejunostomy (P.), 119
Joints, Injuries of, 20
Jones and Castle v. Craunidge, 192
Kidney, Contusions and Lacerations of the (P.), 277
Kidney, Movable (P.), 277
Kilmarnock Infirmary (A. H.), 320
Klein (Dr.) on Antitoxin, 434
Knee. *See Hysterical Knee*
Knee-joint Disease, Etiology of Deformities Occurring in (P.), 349
Kraske's Operation (P.), 279
Lactic Acid in Ulcers of the Cornea (P.), 334
Ladies at Golf, 5
Lateral Sinus Disease (P.), 260
Laparo-Splenectomy for Leukæmic Hypertrophy (P.), 80
Laundries, Hospital (W.), 15
Law Courts, In the. Jones and Castle v. Cranidge, 192
Leeds Union Infirmary Nurses' Home (W.), 85
Leprosy (P.), 47
Lettsomian Lectures, The, 306
Lichen (P.), 82
Liver, Abscess of (P.), 137
Liver, Cirrhosis of the (P.), 241
Liver, Diseases of the (P.), 240
Liver, Hydatid of (P.), 137
Liver, Wound of the (P.), 137
Liver and the Formation of Urea, The (P.), 241
Liver and Gall-bladder, Surgery of (P.), 137, 153
Llangammarch Water as a Heart Tonic (P.), 441
Lockers, Hospital (W.), 372
London Hospital (A. H.), 123
London. Is London Healthy? 291
London Medicine, A New Hope for, 305
Long Bones, Diseases of (in Children) (P.), 294
Lupus (Dermatology) (P.), 101, 368
Lymphangioma (P.), 406
Magnesium Sulphate, Hypodermic use of (P.) 156
Magnet, Removal of Foreign Bodies from the Eye by a (P.), 314
Malignant Growths (C.), 221
"Mallet-Finger" (P.), 349
Malta Fever (P.), 423
Man, The Study of, 93, 165, 325, 379
Marriage or Not Marriage for Women? 290
Medical Congress at Calcutta, The, 280
Medical Education, Some Imperfections of, 290
Medical News from India, 298
Medical Inspection of Schools, The, 145
Medical Journalism and the Public Schools, 363
Medical Literature, English. *See First Century*
Medical Schools, Opening of the, 1
Medical Schools. The Public and the London Medical Schools, 95
Medical and Hospital Progress at Oxford, 181
Medico-Psychology and Psychiatry. *See Modern*
Menière's Disease (Treatment), (P.), 426
"Men's Children," 237
Mental Enfeeblement, Conditions of, 57
Meso-Sigmoid, Sarcoma of the (P.), 120
Methuselah: An Apologue, 327
Metropolitan Asylums Board and Antitoxin Treatment, The, 323
Micro-Organisms of Sewage, 342
Midwifery Muddle, The, 360
Migraine, The Treatment of (P.), 204
Milk Diet in Bright's Disease, 77
Milk Diet and Intestinal Decomposition (P), 330
Mitral Regurgitation (P.), 79
Mitral Stenosis (P.), 79
Modern Medico-Psychology and Psychiatry, 4, 201, 253, 307, 397
Modern Progress in Ophthalmic Medicine and Surgery, On (*and under sub-titles*), 3, 39, 111, 183, 271, 342, 417
Morphine, Large Doses of (P.), 226
Morton's Disease (P.), 350
Muscular Rheumatism (P.), 98
Myomata, The Origin of (Gynæcology), (P.), 352
Nævi (Neck) (P.), 203
Nasal Breathing in Nasal Obstruction (P.), 311
Neck, On the Differential Diagnosis of Swellings in the (C.), 187, 203
Nerve Disorders (P.), 460
Nervous System, Diseases of the (P.), 154, 173, 190
Nervous Diseases and Modern Life, 380
Neuralgia, Trigeminal (P.), 261
Neuritis. The Value of Optic Neuritis in Medical Diagnosis (P.), 97
Neuritis and Neuralgia, 378
Neuroses, Reflex Ocular (P.), 190
New Appliances and Things Medical (W.), 12, 30, 48, 66, 84, 138, 156, 280, 316, 334, 352, 370, 406, 426, 444
New Drugs and Preparations, 30, 83, 102, 174, 226, 262, 297, 315, 334
New Hospital for Women, The (A.H.), 123, 429
Nitrous Oxide (P.), 27
Noises of Great Cities, The, 342
Notes and News: 6, 24, 42, 60, 78, 96, 114, 132, 150, 168, 186, 206, 220, 238, 256, 274, 292, 310, 328, 346, 364, 382, 400, 420, 438, 456
Obstetrical Society and the Medical Council (*and see p.* 382), 287
Ocular Muscles, Diagnosis of Paralysis of the (P.), 315
Œdema, Localised (C.), 347
Œsophagus and Stomach, Surgery of the (P.), 65
Onychomycosis (P.), 81
Ophthalmic Medicine and Surgery. *See Modern*
Ophthalmology (P.), 314
Ophthalmology, Progress in (P.), 347
Optic Neuritis. *See Neuritis*
Optic Thalamus, Tumour of (P.), 174
Orchitis and Parotitis (P.), 296
Osteitis, Septic in Childhood (P.), 295
Osteoclasis for Rhachitic Deformities (P), 350
Otitis Media (P.), 425, 426
Otology (P.), 424
Oyster Scare, The, 273
Paget (Sir J.) on the Scientific Temper, 41
Pamphigus (P.), 102
Panacea in English Medicine, The, 184, 254, 398
Pancreas and Spleen, Surgery of (P.), 80
Pancreatic Cyst (P.), 80
Paper-Jacket, Application of the (P.), 349
Paracentesis Capitis (P.), 25
Paracentesis of the Various Cavities, Notes on the Method of Performing (C.), 7, 25
Paralysis, The Early Symptoms of General, 252
Paralysis, Diphtheritic in Children (C.), 239, 257
Parasitic Diseases (P.), 81, 369
Parotitis and Orchitis (P.), 296
Passive Motion, 2
Pathology (General) of Infective Diseases (P.), 117
Pay System and the Profession, The, 91
Pay System at the Great Northern Central (*and see Great Northern Central*), 363, 389
Pay Wards in General Hospitals (W.), 463
Paying Patients (*and see Great Northern Central*), 372, 387
Perforating Ulcer of Duodenum (P.), 119
Perforation, Intestinal (P.), 119
Perforation of the Uterus from Rapid Dilatation (P.), 351
Pericarditis (P.), 460
Perils of the Post, 360
Periostites Orbital (P.), 222
Peristalsis, The Artificial Production of Reversed (P.), 101
Peritoneal Adhesions, Painful (P.), 443
Peritonitis (P.), 331, 443
Peritonitis, Tubercular (P.), 331, 444
Peritonitis, Treatment of Perforation Peritonitis, 56
Pigmentary Diseases (Dermatology) (P.), 82
Pituitary Body, Functions of (P.), 173
Plague, The (P.), 423 (*and see p.* 46)
Plague of 1167, The, 418
Pleurisy, Syphilitic (P.), 222
Potassium, Permanganate of, as an Antidote (P.), 385.
Practical Departments (W.), 15, 40, 68, 104, 122, 141, 158, 177, 227, 247, 264, 300, 337, 355, 372, 408, 464
Practical Points (W.), 51, 159, 196, 284
Prison as a Reformatory, The, 255
Problem of the Poor, The (*and see p.* 106), 269
Professional Infamy: The Medical Council and the Obstetrical Society (*and see p.* 382), 287
Pruritis (P.), 383
Pruritis, The Electrolytic Treatment of, 219
Psoriasis (P.), 81
Psychologist in History, 22
Puerperal Fever, The Definition of, 414
Pulmonary Circulation, Treatment and The, 361, 435
Pyelo-Nephritis (P.), 276
Pyrexia (P.), 117
Quinine, Action of Some Homologues of (P.), 315
Rabies (P.), 118
Radcliffe Infirmary, Oxford (W.), 139, 196, 248, 282, 292, 301
Rain Baths (W.), 300
Ranulæ (Neck) (P.), 188
Rectal Ailments. *See Anal and Rectal, &c.*
Rectal Mucous Membrane, Superficial Ulceration of the (P.), 384

Rectal Mucous Membrane, Vascular Patches of the (P.), 383
Reflexes, Tendon (P.), 155
Religion in Hospitals, 185
Respiratory Diseases of Children (P.), 276
Retina, Detachment of the (P.), 314
Rheumatic Perisyphlitis (P.), 330
Rolling of the Head, 167
Rotunda Hospital, Dublin, The (W.), 283
Royal Portsmouth, Portsea, and Gosport Hospital (A.H.), 265
Rural Sanitation and the Unemployed, 327
St. Thomas's Hospital (Meeting), 346
Sanitation, Dry, 360
Sanitation: How Sanitation is Done in Ireland, 167
Sane Persons in Private Asylums, 219
Sarcoma of the Meso-Sigmoid (P.), 120
Savill's Disease (P.), 368
Scarlet Fever (P.), 64, 423
Schott: The Schott Treatment of Heart Disease (P.), 99, 441
Scleroderma (P.), 81
Sclerosis, Amyotrophic Lateral (P.), 173
Scopolamine as a Mydriatic (P.), 333
Scraps and Gleanings, 16, 34, 52, 70, 88, 124, 142, 160, 178, 196, 230, 248, 266, 284, 302, 320, 338, 355, 374, 393, 410, 430, 448, 464
Seamen's Hospital, Greenwich, The (A.H.), 160
Seborrhea (P), 81
Serum (*see Therapeutics*), 127
Sewage Farming in India, 345
Should First Cousins Marry? 455
Skin Grafting and Allied Procedures (C.), 293
Small-pox (P.), 135
Social Problems, 416
Sociology, Modern, 308, 362, 436
Sociology, Studies in Applied, 58, 94, 130, 218
Stenosis: Mitral Stenosis (P.), 79
Song or a Sermon, A, 291
Spasmotin or Sphacelotaxin (P.), 297
Speech Centres (Nervous Diseases) (P.), 190
Spina Bifida (P.), 262
Spinal Caries and Its Treatment (P.), 349
Spinal Cord, Hæmorrhagic Necrosis of (P.), 173
Spine, Injuries to the (P.), 261
Spine, Malignant Disease of the (C.), 311
Spleen, Solitary Hydatoid Cysts of the (P.), 80
Spring Headaches, 455
Stenosis, Mitral (P.), 79
Story of the Insane from Year to Year, The (W.), 14, 33, 51, 176, 264, 317, 337, 464
Student of Medicine, The (Appointments, Vacancies, Notes), 17, 36, 53, 71, 89, 107, 125, 143, 161, 179, 197, 231, 249, 267, 285, 303, 321, 339, 357, 375, 393, 411, 431, 449, 467
Strychnine (Hypodermic Use of) (P.), 10
Sub-Nitrate of Bismuth, Poisoning from (P.), 244
Sub-Umbilical Space, Acute and Chronic Abscess of the (P.), 29
Sub-Umbilical Space, Rupture of Abscesses into the (P.), 29
Sub-Umbilical Space, Tumours of the (P.), 29
Sunderland, New Eye Infirmary (W.), 407
Surgeons and Non-Qualified Men, 192
Surgery (P.), Abdominal, 28, 443
Surgery of the Bladder (P.), 242
Surgery, Cranial, 259
Surgery, General, 11, 222, 242, 312, 332
Surgery, Genito-urinary, 222, 460
Surgery of the Intestines, 100, 119
Surgery of the Liver and Gall Bladder, 137, 153
Surgery of the Œsophagus and Stomach (P.), 65
Surgery, Orthopædic, 348
Surgery of the Pancreas and Spleen, 80
Surgery, Rectal, 278, 296
Surgery, Renal, 276
Surgery, Spinal, 261
Surgery of the Vermiform Appendix, 171
Sweet Charity's Guide for Christmas Givers: General Hospitals, 207; Special Hospitals, 209; A few other Charities, 211
Syphilis (P.), 222
Syphilis of the Epididymis, 222
Syphilis of Pharynx, 222
Syphilis Hereditary in Children (P.), 295
Syphilitic Pleurisy (P.), 222
Syringomelia (P.), 164
Tadpole's Tail, Disappearance of the, 185
Talipes, Practical Points in the Treatment of (P.), 349
Tamponning (Gynæcology) (P.), 350
Tannigen; an Intestinal Astringent (P.), 334
Tapeworm, Treatment of (P.), 311
Tea, Dangers of Strong, 131
Teaching University, A, a New Hope for London Medicine, 305
Testicle, Strangulation of the (P.), 243
Tetanus, Treatment of, by Serum (P.), 424
Text-book Surgery, 113
Therapeutic Notes (P.), 370, 385
Therapeutics, The Future of Serum, 129
Therapeutics and the Lesser Circulation, 147, 217, 289, 361, 435
Thyroid Feeding (P.), 101, 406
Thyroid Tumours (P.), 203
Tibia, Congenital Absence of the Entire (P.), 349
Tonsillitis (Puerperal Temperature) (P.), 151
Tottenham Hospital, The Religious Question at the, 455
Toxic Neutralisations, On, 21, 75
Toxicological Memoranda (P.), 156
Toxicological Note (P.), 244
Trachoma, Surgical Treatment of (P.), 333
Tracheotomy, 324
Trephining (P.), 259
Trikresol (P.), 156
Tsar, Illness of the, 5
Tsar's Illness and its Lessons, The, 110
Tsar's Kidney Affection, The, 59
Tuberculosis (Spinal Surgery) (P.), 261
Tuberculosis in Cattle, 95
Tuberculosis, Anal (P.), 297
Tumours, Fatty (Neck) (P.), 203
Tumours of Muscles of the Neck (P.), 203
Tumours, Removal of (P.), 259
Tympanic Vertigo, Chronic (P.), 426
Typhoid (P.), 223
Ulnar Nerve, Traumatic Paralysis of (C.), 152, 170
Unqualified Practice at Hospitals, 128
Urticaria (P.), 369
Ureter, Calculus of the (P.), 242
Uterus, Ventro-fixation of the (C.), 43
Uterus (*see Perforation*), 351
Uterus, Backward Displacement of the (P.), 351
Vacancies (*The Student of Medicine*)
Vaccination Question, The (C.), 421, 439
Vaginal Douching (Editor's Letter-Box), 370
Valvular Lesions (Heart Diseases) (P.), 79
Varicose Veins, Surgery of (P.), 12
Ventro-fixation of the Uterus (C.), 43
Vermiform Appendix, Surgery of the (P.), 171
Walnut Lodge Hospital, Hartford, Ct. (W.), 227
West Sussex, East Hants, and Chichester General Infirmary (A. H.), 320
Wholeman Specialism, 363
Within the Asylums (W.), 86
Within the Hospitals (W.), 103, 121, 139, 175
Women, Causes of Diseases Peculiar to (P.), 351
Workshops and Drink, 433
Wound Infection, 396
Wry-Neck (P.), 348

### THE SPECIAL SUPPLEMENT FOR NURSES.

Addenbrooke's Hospital, Cambridge (*and see p.* xxi), xxvii, cxi, cxliii
Albany, The Duchess of, xxxiii
American Letter, Our, xx, xliii, xcv, cxxi, cl, clxxv
American Nursing, An English View of, cxxxii
American Woman at Home, The, xlv, xlix, lix, lxxv, civ
Anatomy and Surgery for Nurses, Elementary, cxi, cxvii, cxxvii, cxxxvii, cxliii, cxlix, clv, clxi, clxvii, clxxiii, cxci
Anerley, An Action at, clxxvii
Antiseptics, lvii
Appointments, x, xx, xxxii, xxxvii, xliv, liii, lxii, lxiii, xc, civ, cxliv, cxlix, clvi, clxii, clxxx, clxxxiv, cxciv
Appointments, Minor, xx, xxxvii, xlix, liv, lxii, lxxxviii, cvi, cxxxviii, clxiii, cxcii
Army Sisters, v
Asylum News and Nursing, xxxiv, lviii, cxxix, clxii
Begging Letter Writers, cxlvi
Book World for Women and Nurses, The, cxxxix, cxlvi, clii, clviii, clxx
Burdett's Official Nursing Directory, clvii, clxiii, clxvii
Cairo, Notes from, cxliv
Christmas Books, lxxxv, xcviii
Christmas Competition, Our, xciv
Christmas Fare Two Centuries Ago, lxxix
Christmas Number, Our, lxxiii
Christmas, Present for, A, lxxiii
Christmas, How, When, and Where Nurses Spend, lxxxi
Christmas on an Australian Fruit Farm, lxxxiv
Christmas in Germany, cvi
Christmas in an Irish Hospital; lxxxviii
Christmas and New Year's Gifts, lxxxix
Convalescent Fund, The Hospital, iii
Countess of Dufferin's Fund, xiv
Death in our Ranks, xxxvii, lxx, clxii, clxxvi, cxcii
Dress and Uniforms, vi, xvi, xxii, xxviii, xl, xlvi, lii, lxviii, c, cxii, cxxx, cxliv, cli, clxxvi
East London Nursing Society, clxxxiv
Edmonton Workhouse, A Visit to, ix
Entertainments, xciv, cvii, cxiii, cxxiii
Everybody's Opinion, xv, xxi, xxvii, xxxiii, xl, lxx, lxxvi, xc, xcvii, cvi, cxxiv, cxxxiv, cxl cxlvi, cl, clv, clxiv, clxix, clxxx
Fever Hospitals, Nursing in Metropolitan, v
French Schools for Trained Nurses, xliii, cxxxiii
From Two to Three Guineas, lxix
Germany, Notes from, l, cxxxix
Glasgow System for Probationers, The, xxxix
Glasgow Western Infirmary, cxix
Great Movement, A: The Nurses' Co-operation, cxciii
Help the Nurses to Help the Sick, lxxxiii
Helps Homewards, cxx
Holland, Notes from, cxcii
Home Nursing, Hints for, clxxvi
Hong Kong, The Government Civil Hospital, lxvi, cxxxi
Hostel of God, The, cxl
India, Notes from, lxix, lxxiv, cxiv
Infancy, The Dietetic Management of, lxv, lxxiii, xciii, ciii
Lambeth Infirmary, The, cxx
Lambeth Workhouse Infirmary, The Training of Probationers at, cxiv
Legal Intelligence, clvii
Lewisham Infirmary, clvi, clxiii, clxxvi, clxxxiii
Lying-in Hospital, Pupils in a, liv
Madhouse of Fiction, The, cxvii
Male Nurses, x, xxvi
Male Nurses' Temperance Co-operation, xxxiv
Marriages, xiv, xlv
Massage, x
Matrons' Council, The, xxxix
Matrons in Council, xxvi
Meath Hospital and Co. Dublin Infirmary, clviii
Metropolitan Hospital, The (Concert), cxciii
Muses' Looking Glass, The, cxxii
Natal, Hospitals in, liii
National Pension Fund for Nurses, The Royal, clxxiv, clxxxvi
New Society, A, xxv
New South Wales, xiv
News from the Nursing World, i, xi, xvii, xxiii, xxix, xxxv, xli, xlvii, lx, lxiii, lxxvii, xci, ci, cix, cxv, cxxv, cxxxv, cxli, cxlvii, cliii, clix, clxv, clxxi, clxxxi, cxc
Notes from St. Helena, xxxvii
Notes and Queries, xi, xxviii, xxxiv, xxxix, xliv, lxix, lxxxiv, cxiv, cxxii, cxxxiii, cxl, cxlv, cl, clvi, clxiv, clxviii, clxxiv, clxxxviii
Notes for Nurses on Antiseptic Surgery, xiii, xxv, xxxi
Novel Competition, A, viii
Novelties for Nurses, x, clxii
Nurse and the Sister, The, clxxix
Nurses as Sanitarians, xix
Nurses as Stewardesses, Trained, x
Nurses, English, at Hong Kong, clvi
Nurses, Mental, v
Nurses, Old Time, lxi, lxvii
Nurses, Scattered, iv
Nurses' Co-operation, The, cxciii
Nursing Directory, Burdett's Official, clvii, clxiii, clxvii
Nursing at Addenbrooke's Hospital, xxi
Nursing in Cairo, clxix
Nursing in Dublin cxxiii
Nursing in Germany, xxxii
Nursing in Holland, xcvii, clxxx
Nursing in Holland, District, lxxix
Nursing at Home and Abroad, Private, xcvi
Nursing in Ireland, xliv, lx
Nursing in Japan, cxxviii
Nursing on the Labrador, lxii
Nursing in Metropolitan Fever Hospitals, v
Nursing in Military Hospitals, lv, lxvi
Nursing in New South Wales, clxii
Nursing in Rio de Janeiro, xx, xxxvii, cxx
Nursing in the U.S.A., xxxii
Nursing in the West Indies, cxlv
Nursing in Workhouses, v
Orphans in Cages, lx
Orphans Still Caged, cxliv
Our Work, iii
Pension Fund for Nurses, Royal National, clxxiv, clxxxvi
Poor of Spitalfields, The, xxxi
Poor Law, The Humanising of the, cxxxviii
Presentations, vii, xxvii, xxxii, xxxix, lvii, lxx, lxxv, cvi, cxiii, cxxii, cxlv, clv, clxviii, clxxxiv
"Princess, Our," lxxx
Probationers and Junior Nurses, cl
Queen Victoria's Jubilee Institute, cxiii
Reading to the Sick, For, xcviii, cxxxiv, clii, clxx
Royal British Nurses' Association, xxxii, lxix, lxxiv, clvi, clxxvi, clxxxiii
Royal National Pension Fund for Nurses, clxxiv, clxxxvi
Saint Helena, Notes from, xxxvii, liii, xcv, cxl
Should Fever Nurses be Trained? lxii
South-Eastern Hospital, New Cross, cxxxi
Spring Novelties, cxxxviii
The Hospital Convalescent Fund, iii
"Truth" Toy Competition, xcvi
Victoria, News from, xxxviii
Wants and Workers, x, xxviii, xxxviii, xliv, liii, lx, lxxv, ciii, cxiv, cxix, cxxxiii, cl, clxiv, clxxiii, clxxxiv, cxcii
Where to Go, v, xv, xxxix, xlvi, liv, lvii, lxv, lxxiv, lxxxviii, cxiv, cxxii, cxxxii, cxlvi, cl, clxii, clxvii, clxxx
Wigmore Co-operative Nursing Institution, x
Workhouse Infirmary Nursing Association, lxii

Printed by W. H. and L. Collingridge, "City Press," 148 and 149, Aldersgate Street, London, E.C.

**Notices of Births, Deaths, and Marriages** are inserted in this column at a charge of 2s. 6d. for an announcement not exceeding four lines.

**Notice to Advertisers and Subscribers.**

All Advertisements, Orders for Copies of The Hospital, and Business Communications should be addressed to **The Publisher, NOT TO THE EDITOR,** The Hospital, 428, Strand, W.C.

The Cost of Subscription, post free, is as follows:—Per week, 2½d.; per quarter, 2s. 8d.; per half-year, 5s. 3d.; per annum, 10s. 6d. Foreign:—Per Annum, 12s. 8d.; payable, in all cases, in advance.

### NOTICE TO CORRESPONDENTS.

All MS., Letters, Books for Review, and other matters intended for the Editor should be addressed The Editor, The Lodge, Porchester Square, London, W.

The Editor cannot undertake to return rejected MS., even when accompanied by stamped directed envelope.

**"Burdett's Hospital Annual," 1894.**

Fifth year of publication. 900 pp. Boards, gilt lettering. A most complete guide to British, Colonial, Indian, and American Hospitals, Dispensaries, Nursing and Convalescent Institutions, and Asylums. Price 5s. Published by The Scientific Press (Limited), 428, Strand, W.C.

**NOTICE.—The Index for Vol. XV. (ending March 31st, 1894) is on sale at the Office of this Journal, price 2½d., post free.**

### Notes and News.

### SCRAPS AND GLEANINGS.

A medical school for women is about to be established in St. Petersburg under government auspices.

The annual appeal on behalf of the funds of Leicester Infirmary has proved this year to be the best on record.

The 24th annual general meeting in connection with the Newcastle Hospital Sunday Fund showed in its report a decrease in the contributions.

The North Cambridge Hospital and Hunstanton Convalescent Home have benefited to the extent of £28, through the auspices of the annual Hospital Sunday scheme at Wisbech.

A new wing has been added to the Heatherdene Convalescent Home, an adjunct of the Sunderland Infirmary. The ceremony took place on the second anniversary of the foundation of the home.

The Hospital Saturday collection in aid of the Wolverhampton and South Staffordshire General Hospital have proved successful this year. The street collections alone resulting in over £74 being raised.

Hospital Sunday collections, organised by the friendly societies of Leighton, have just been made in favour of the funds of University College and St. George's Hospitals, London, the Aylesbury Infirmary, and the Leighton Dispensary.

A house-to-house canvass was made recently at Orpington and in the Crays under the auspices of the Pride of Kent Lodge of Oddfellows, in aid of the Cray Valley and Chislehurst Cottage Hospital and the Friendly Societies' Convalescent Home at Dover.

A bazaar was held this month at West Kirby in favour of funds for the fitting and maintenance of the new wing of the Convalescent Home of that locality. The committee report another gift of a cheque for £100 from Mr. H. J. Woolcott, of Birkenhead.

An earnest appeal is being made in favour of the funds of the Harrogate Bath Hospital and Rawson Convalescent Home, an institute which annually benefits upwards of 11,000 patients. Donations are asked to help wipe off a debt of £1,139.

A corps of trained female nurses has been despatched from the Tokio training school to the military hospital at Seoul, and others are being trained for this service. It is said that ladies of the highest rank have offered their services as nurses at the seat of war.

An extraordinary medical syndicate is reported from Iowa (America). This is the scheme of assessing every church member 50 cents a month, in return granting them free medical attendance and three dollars a week during illness, and the entire funeral expense in case of death.

Protests continue to be urged against the erection of a permanent smallpox hospital at Mode Wheel (Salford). The residents in the Weaste district consider a more isolated position should be secured for the building than the one which has already been suggested in their midst.

A large "At Home" has just been given at the Aberdeen Hospital for Incurables. The committee of management, who bore the cost of the entertainment, organised the scheme with the hope of enlisting further the local sympathy with this most deserving institution.

Lord Ducie has forwarded a cheque for £500 to the Gloucestershire Infirmary and Eye Hospital. This bequest, he explains, was contained in his will, but as the sum would be diminished by charges made by the State, after his death, he thinks it right to make over the money during his lifetime.

Notwithstanding that it was a new movement, and had been preceded by an equally successful lifeboat demonstration, it was most gratifying to the promoters to know that the result of the Hospital Saturday movement in Blackpool had resulted in the addition of no less than £300 to the funds of the local institution.

The Holborn Restaurant is a popular resort for hospital festivals. Here a dinner of unique character was given on Saturday night to a company numbering 600, in the palatial new annexe of the Restaurant. The guests consisted of all the artificers and workmen employed in the construction of the same.

The Amalgamated Local Friendly Societies are doing a public service at Chesterfield by calling attention to the need existing at the Chesterfield and North Derbyshire Hospital for the treatment of medical cases within its wards, space and funds hitherto having proved deterrent reasons against this provision.

A fete has been held in the grounds of the Corbett Hospital, Stourbridge, by the friendly societies of the district. The Committee have handed over to the hospital the sum of £124, being the proceeds of this scheme. Some further small sum has been withheld in view of the expenses connected with the fête next year.

Since the Hospital Saturday Fund has been organised at Exeter, something like £9,500 has been collected. The Devon and Exeter Hospital has rather an uphill struggle to make its annual expenditure square with the income, and much good work has been done by the members of the various local friendly societies to increase the finances.

In consequence of the increased demands made upon the already extensive accommodation of the Wolverhampton Hospital for women, the committee decided to increase the same by taking in an adjoining house. This extension has now been completed, which will be the means of making the hospital even more efficient than before.

Belfast is to be congratulated on the important step taken to raise a building fund for the New Mater Infirmorum Hospital. The subscriptions towards which institution, most of which were handed in at the meeting, reach close on £5,000. Belfast, as compared with other cities, is far behind in hospital accommodation, and this step is one in the right direction.

The proceeds resultant on the annual Demonstration of the Friendly Societies at Chelmsford was divided in the following grants: The Clacton Convalescent Home benefiting by £45, the Chelmsford Infirmary and Dispensary by £25, and £10 was contributed to the Ladies' Committee for the purchase of some suitable article of furniture for the use of invalids at the first-mentioned infirmary.

### THE STUDENT OF MEDICINE.

### APPOINTMENTS.

C. R. Edmonson, M.B., C.M.Edin., appointed Second House Surgeon to the Royal Southern Hospital, Liverpool. Mr. W. P. Fooks, appointed Assistant Medical Officer to the Workhouse and Infirmary of the Parish of Paddington. R. G. Hogarth, F.R.C.S.Eng., L.R.C.P.Lond., appointed Junior Resident Medical Officer to the Nottingham General Hospital. John Larwill, L.R.C.P.Edin., L.R.C.S.Edin., L.F.P.S.Glasg., appointed Junior House Surgeon to the Macclesfield General Infirmary. J. Marsh, M.B., C.M.Edin., appointed Junior House Surgeon to the Royal Southern Hospital, Liverpool. John Alfred Waring, M.B., L.R.C.P.Lond., M.R.C.S.Eng., appointed Senior Resident Medical Officer to the Nottingham General Hospital. Alfred Woodward, M.R.C.S.Eng., reappointed Medical Officer of Health to the St. Helen's Urban Sanitary District. J. Young, M.D.Glasg., reappointed Medical Officer of Health to the St. George's Urban Sanitary Authority.

### VACANCIES.

The following vacancies are announced this week, the amount of salary in each case being given after the name of the institution:

#### Resident Medical Officers.

Ancoats Hospital, Manchester.—Resident Junior House Surgeon. Salary £60 per annum, with board and washing. Must be duly qualified and on the Medical Register. Applications and testimonials to A. Forrest, Honorary Secretary, before October 10th.

Cumberland Infirmary, Carlisle.—Assistant House Surgeon. Salary £40 per annum, with board, lodging, and washing. Appointment for one year. Applications to the Secretary by October 9th.

East London Hospital for Children, Glamis Road, Shadwell, E.—House Physician. Board, lodging, &c., provided, but there is no salary attached to the post. Applications to the Secretary by October 6th.

General Infirmary and Dispensary, Doncaster.—Indoor Dispenser and Assistant to House Surgeon. No salary, but board, lodging, and washing provided. Applications and testimonials to J. Clark, Honorary Secretary, before October 9th.

General Hospital, Birmingham.—Resident Surgical Officer and House Physician. Salary £130 and £70 per annum respectively, with residence, board, and washing. Appointments for one year, but candidates will be eligible for re-election for two subsequent years. Also two Assistant House Surgeons, appointment for six months, but eligible for re-election for another six months. No salary. Residence, board, and washing provided. Applications to Howard J. Collins, House Governor, by October 6th.

Hospital and Dispensary, Newark-upon-Trent.—House Surgeon. Salary £80 per annum. Will be required to do the dispensing, for which a further salary of £40 is paid. Applications and testimonials to W. Easterfield, Secretary, by October 6th.

London Fever Hospital, Liverpool Road, N.—Assistant Resident Medical Officer. Salary £120 per annum. Applications to the Secretary by October 8th.

National Hospital for Diseases of the Heart and Paralysis, Soho Square. —Resident Medical Officer; must be doubly qualified. Appointment for six months; residence in the Hospital and an honorarium of £10 10s. Applications and testimonials to Capt. F. Hanaley, Secretary, before October 13th.

Rotherham Hospital and Dispensary.—Assistant House Surgeon. No salary, but board, lodging, and washing provided. Appointment for six months. Applications and testimonials to the House Surgeon before October 18th.

Royal Albert Hospital, Devonport.—Assistant House Surgeon for six months. Board, lodging, and washing provided. No salary. Applications to Chairman of Medical Committee by October 10th.

#### Non-Resident Medical Officers.

Dental Hospital of London, Leicester Square.—Assistant Dental Surgeon. Must be Licentiate of Dental Surgery of one of the licensing bodies recognised by the General Medical Council. Applications and testimonials to J. Francis Pink, Secretary, by October 8th.

General Infirmary at Gloucester and Gloucestershire Eye Institution.—Assistant Surgeon; must be F.R.C.S. or M.R.C.S.Eng., or F.R.C.S. or L.R.C.S. of Ireland or Edinburgh, or graduate of University recognised by the General Medical Council. Applications and testimonials to Henry T. Pike, Secretary, by October 10th.

THE STUDENT OF MEDICINE—*continued.*

St. Olave Board of Works.—Medical Officer of Health for the District; not less than 25 or more than 45 years of age; must reside in or within one mile of the district. Salary £200 per annum. Applications to Edric Bailey, Clerk, Vine Street, Tooley Street, S.E., by October 15th.

Wandsworth Board of Works.—Medical Officer of Health for the Parish of Wandsworth; must reside within the parish. Salary £150 per annum, rising, subject to the order of the Board, by annual increments of £10 to £200 per annum. Applications, endorsed "Medical Officer," on forms to be obtained of the Clerk, to be sent to the Board's Offices, East Hill, Wandsworth, by October 8th.

### UNIVERSITIES AND COLLEGES.

### University of Durham.

EXAMINATION FOR DEGREES IN MEDICINE AND SURGERY.

The following candidates have satisfied the examiners:—

Second Examination for the Degree of Bachelor in Medicine. First Class Honours: E. Turner, M.R.C.S., L.R.C.P., St. Bartholomew's Hospital. Second Class Honours: C. Salkeld, B.A., College of Medicine, Newcastle-upon-Tyne; A. Riley, Westminster Hospital. Pass List: A. W. Aldridge, Mason College, Birmingham; M. Archdall, College of Medicine, Newcastle-upon-Tyne; G. Arnott, College of Medicine, Newcastle-upon-Tyne; H. E. M. Baylis, St. Bartholomew's Hospital; C. H. Clarke, Mason College, Birmingham; H. A. Crossfield, St. Thomas's Hospital; J. D. Dodds, College of Medicine, Newcastle-upon-Tyne; J. L. Frazer-Hurst, College of Medicine, Newcastle-upon-Tyne; J. R. Fuller, M.R.C.S., L.R.C.P., L.S.A., St. Mary's Hospital; E. W. Gilroy, College of Medicine, Newcastle-upon-Tyne; H. J. Godwin, St. Bartholomew's Hospital; H. H. Gourley, Edinburgh University; W. Hodges, St. Thomas's Hospital; P. W. James, St. Bartholomew's Hospital; J. Lowry, College of Medicine, Newcastle-upon-Tyne; M. G. McBean, College of Medicine, Newcastle-upon-Tyne; N. McBall-Smith, College of Medicine, Newcastle-upon-Tyne; M. S. Paterson, M.R.C.S., L.R.C.P., St. Mary's Hospital; T. O. Scott, M.A., College of Medicine, Newcastle-upon-Tyne; H. F. Shea, St. Thomas's Hospital; G. Stonehouse, College of Medicine, Newcastle-upon-Tyne; E. W. Sumpter, College of Medicine, Newcastle-upon-Tyne; W. A. H. Waite, Yorkshire College, Leeds; W. L. W. Walker, College of Medicine, Newcastle-upon-Tyne; W. H. Warwick, College of Medicine, Newcastle-upon-Tyne; J. H. Wood, College of Medicine, Newcastle-upon-Tyne.

### University of St. Andrews.

Her Majesty's Commissioners for the Exhibition of 1851 have placed a recommendation to a science research scholarship of the annual value of £150 at the disposal of the University of St. Andrews for the year 1895.

It was reported on September 22nd to St. Andrews University Court that the Commissioners of Her Majesty's Treasury intimated that it is intended to repeal the stamp duty hitherto payable on the degrees of M.D.

### Society of Apothecaries of London.

PASS LIST, SEPTEMBER, 1894.

The following candidates passed:—

Surgery.—S. H. L. Archer, London Hospital; G. Candler, St. Thomas's Hospital; J. W. Clegg, Manchester; H. C. Dixon, London Hospital; T. Morris, Liverpool; L. E. V-é-tua Saville, Royal Free Hospital; G. A. Skinner, Guy's Hospital; M. Umanski, Kharcoff; J. C. V. Wilkins, Guy's Hospital.

### Medical Appointments.

NATIONAL HOSPITAL DISEASES OF THE HEART AND PARALYSIS, SOHO SQUARE.

